# Biomimetic scaffold‐based stem cell transplantation promotes lung regeneration

**DOI:** 10.1002/btm2.10535

**Published:** 2023-05-05

**Authors:** Linjie Wang, Meng Feng, Yazhen Zhao, Bing Chen, Yannan Zhao, Jianwu Dai

**Affiliations:** ^1^ Center for Disease Control and Prevention of People's Liberation Army Beijing China; ^2^ Institute of Combined Injury, State Key Laboratory of Trauma, Burns and Combined Injury, Chongqing Engineering Research Center for Nanomedicine, College of Preventive Medicine, Chongqing Engineering Research Center for Biomaterials and Regenerative Medicine Army Medical University, Third Military Medical University Chongqing China; ^3^ State Key Laboratory of Molecular Developmental Biology Institute of Genetics and Developmental Biology, Chinese Academy of Sciences Beijing China

**Keywords:** artificial lung ECM‐mimicking scaffolds, clinical‐grade mesenchymal stem cells, lung regeneration, partial lung resection

## Abstract

Therapeutic options are limited for severe lung injury and disease as the spontaneous regeneration of functional alveolar is terminated owing to the weakness of the inherent stem cells and the dyscrasia of the niche. Umbilical cord mesenchymal‐derived stem cells (UC‐MSCs) have been applied to clinical trials to promote lung repair through stem cell niche restruction. However, the application of UC‐MSCs is hampered by the effectiveness of cell transplantation with few cells homing to the injury sites and poor retention, survival, and proliferation in vivo. In this study, we constructed an artificial three‐dimensional (3D) biomimetic scaffold‐based MSCs implant to establish a beneficial regeneration niche for endogenous stem cells in situ lung regeneration. The therapeutic potential of 3D biomimetic scaffold‐based MSCs implants was evaluated by 3D culture in vitro. And RNA sequencing (RNA‐Seq) was mapped to explore the gene expression involved in the niche improvement. Next, a model of partial lung resection was established in rats, and the implants were implanted into the operative region. Effects of the implants on rat resected lung injury repair were detected. The results revealed that UC‐MSCs loaded on biomimetic scaffolds exerted strong paracrine effects and some UC‐MSCs migrated to the lung from scaffolds and had long‐term retention to suppress inflammation and fibrosis in residual lungs and promoted vascular endothelial cells and alveolar type II epithelial cells to enter the scaffolds. Then, under the guidance of the ECM‐mimicking structures of scaffolds and the stimulation of the remaining UC‐MSCs, vascular and alveolar‐like structures were formed in the scaffold region. Moreover, the general morphology of the operative lung was also restored. Taken together, the artificial 3D biomimetic scaffold‐based MSCs implants induce in situ lung regeneration and recovery after lung destruction, providing a promising direction for tissue engineering and stem cell strategies in lung regeneration.

## 1 INTRODUCTION

End‐stage lung disease remains the leading cause of death in the industrialized world owing to the complex nature and slow regeneration capacity of the lungs.^1^ Unlike tissues with a rapid renewal capacity, which have well‐defined hierarchies of stem cells and differentiated cells, the mature lung of mammals maintains homeostasis with a slow cellular turnover, and regeneration and renewal of the lung depend on multiple cell populations.^2^, ^3^ The stem/progenitor cell populations in the trachea, bronchioles, and alveolar contribute to lung maintenance and perform regeneration and repair upon injury, but the dyscrasia of niche in the injured area is not sufficient to continuously support the slow regeneration process, resulting in the failure of repair.^4^ Thus, a beneficial regenerative microenvironment for endogenous stem cells is vitally necessary for situ lung regeneration.

Mesenchymal stem cells (MSCs) have emerged as an attractive measure for regenerative medicine.^5^, ^6^ Umbilical cord‐derived MSCs (UC‐MSCs) are a promising MSC source for clinical cell‐based treatments because of their strong paracrine effects, immunomodulation, high proliferative potential, low immunogenicity, abundant supply, and painless collection.^7^, ^8^ There is growing evidence that UC‐MSCs play a vital role in repairing multiple tissues such as peripheral nerves, skin, bones, kidneys, liver, brain, and endometrium.^9^, ^10^, ^11^, ^12^, ^13^, ^14^, ^15^ In lungs, considering the complexity in terms of structural and cellular diversity, the unique ability of UC‐MSCs to interact with multiple cell types to maintain and promote the survival of injured cells makes them a promising approach for lung regeneration.^16^, ^17^, ^18^ It has been reported that the use of UC‐MSCs and/or their secretome as a therapeutic strategy to improve lung transplantation. Although the mechanisms are not fully defined yet, it has been widely demonstrated in both preclinical and clinical studies that the therapeutic effects of MSCs are mediated to mitigate fibrosis, inflammation, ischemia–reperfusion injury, bronchiolitis obliterans syndrome, and promote the repair of residual endogenous lung stem/progenitor cells, and improve ex vivo lung perfusion during lung transplantation.^19^, ^20^, ^21^ Emory University (ClinicalTrials.gov Identifier: NCT01668576) and Mayo Clinic in Florida (ClinicalTrials.gov Identifier: NCT02181712) have completed the Phase 1 clinical trials of UC‐MSCs on lung transplant and rejection for the safety and intervention dose test. And Department of Cardiology, The Heart Centre, University Hospital Rigshospitalet (ClinicalTrials.gov Identifier: NCT04714801) is also conducting a clinical trial on “MSCs in Lung transplantation”, and is in the recruiting phase. These clinical trials confirmed the potential of UC‐MSCs for lung repair. To be clear that UC‐MSCs do not differentiate into lung tissue but rather exert paracrine effects on epithelial cells, immune cells, endothelial cells, and endogenous stem cell populations for lung repair following injury.^22^ These paracrine effects are mediated by factors such as hepatocyte growth factor (HGF) and vascular endothelial growth factor (VEGF), which are closely related to lung immunomodulation, epithelial stem cell niches, and angiogenesis.^23^, ^24^, ^25^, ^26^ However, the application of UC‐MSCs is hampered by the effectiveness of cell transplantation with poor cell retention, survival, and proliferation in vivo.^27^ In fact, only 1%–20% of transplanted cells survive, which restricts the clinical therapy potential.^28^

Decellularized lung scaffolds are one representative approach to artificial whole lung scaffolds. In a previous study, a decellularized lung scaffold seeded with epithelial and endothelial cells was perfused with air and blood, which generated gas exchange in a bioreactor. When implanted into mice, this artificial lung system provided gas exchange for a short period.^29^ Various procedures to construct lung acellular scaffolds have been investigated, which extended the time of gas exchange. Among the decellularization processes, collagen was largely preserved, resulting in the loss of other extracellular matrix proteins such as glycosaminoglycans and elastin.^30^ Therefore, collagen might play an essential role in lung regeneration. Collagen as the main component of the extracellular matrix is a kind of natural biomaterial that has been widely used for tissue engineering scaffolds in the field of tissue regeneration because of its biodegradability, low antigenicity, and good biocompatibility.^31^, ^32^ A collagen scaffold not only supports the framework of tissue but also regulates cell adhesion, migration, and differentiation.^33^ In past studies, collagen scaffolds loaded with MSCs have reduced the loss and death of MSCs and were applied to the regeneration of other tissues including nerve, endometrium, heart, and bone.^34^, ^35^, ^36^, ^37^

**SCHEME 1:**
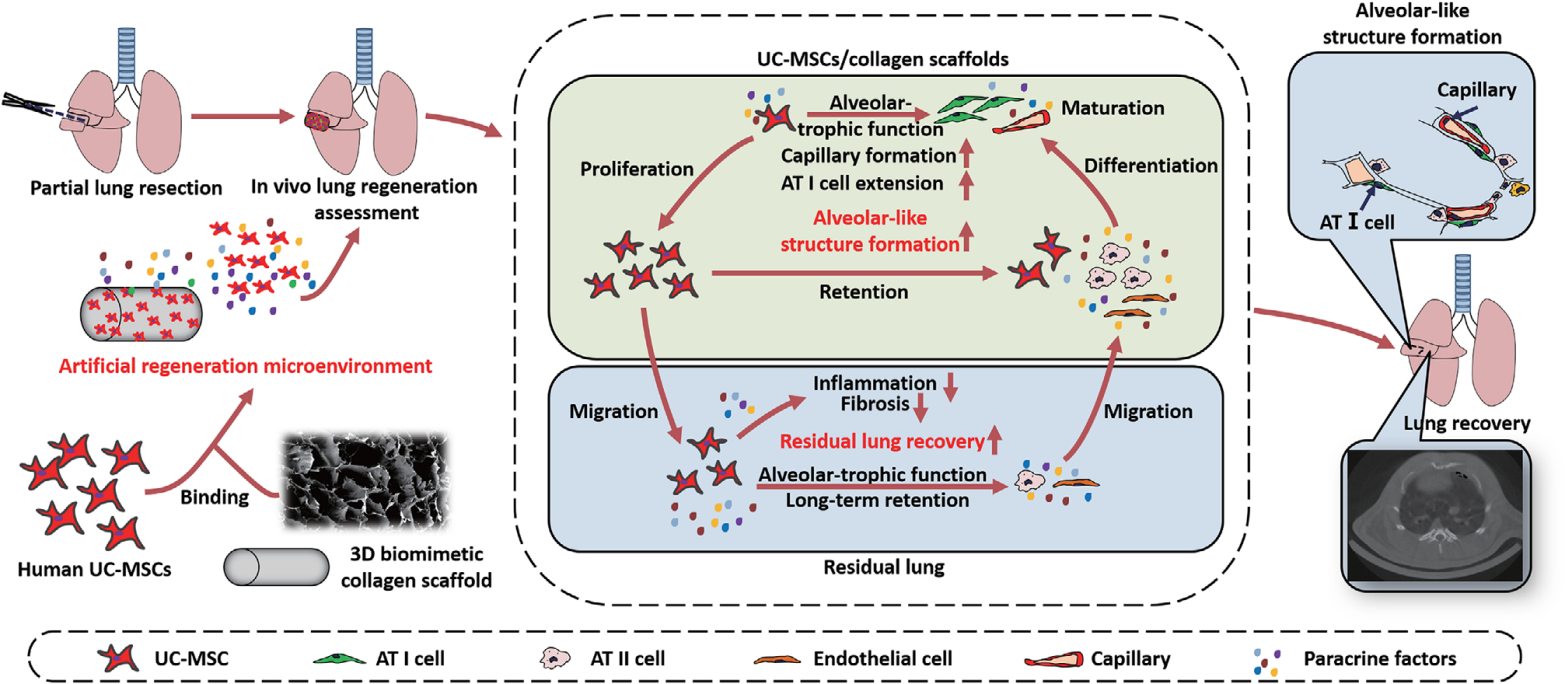
Schematic representation of the prepared 3D biomimetic scaffold‐based MSCs implants and the beneficial niche to drive the behaviors of alveolar stem cells (AT II cells) and endothelial cells to promote alveolar‐like structure regeneration and residual lung recovery.

In the present study, we prepared a porous three‐dimensional (3D) collagen scaffold with pore diameters ranging from 50 to 100 μm, which had similar structure characteristics to the lung. Then, passage three UC‐MSCs were uniformly loaded on the 3D collagen scaffold. Thus, an artificial stem cell niche was established. Next, the collagen/UC‐MSCs scaffold was implanted orthotopically into a partial lung resection model. The results demonstrated that collagen/UC‐MSCs scaffold exerted strong paracrine effects in vitro, promoted the behaviors of alveolar stem/progenitor cells and endothelial cells, suppressed inflammation and fibrosis, restored the general morphology, and formed alveolar‐like structures in vivo, suggesting a clinical potential for lung repair (Scheme 1).

## 2 MATERIALS AND METHODS

### 2.1 Ethics and human UC‐MSCs isolation‐culture

Human umbilical cord tissue was obtained from full‐term fetuses after normal vaginal delivery at the Obstetrics Department of the Southwest Hospital. The study was approved by the Animal Ethics Committee of the Institute of Genetics and Development, Chinese Academy of Sciences. And the approved protocol number is IGDB‐2018‐IRB‐007. Particularly, the object of this study was rats, and did not harm the donor's health. And to protect the privacy of donors, informed consent was obtained from the parents and families, and it was a single‐blind trial for us, where the sex of the umbilical cord was randomized. In this series of experiments presented below, we detected the karyotype of the cell line, suggesting that the sex of the cell line was male. The procedure for UC‐MSCs isolation was carried out according to our previous study.^5^ The isolated UC‐MSCs were maintained at 37°C in a 5% CO_2_ humidified atmosphere with LG‐DMEM/F‐12 medium (11330032, Gibco, USA) supplemented with 10% fetal bovine serum (10099‐141, Gibco, USA), 100 IU/mL penicillin, and 100 μg/mL streptomycin. Human UC‐MSCs at passages three to five were used for the following experiments.

### 2.2 Flow cytometric analysis

Flow cytometric analysis of human UC‐MSCs surface antigens was performed at the fourth passage. First, 1 × 10^6^ cells were incubated with 1% bovine serum albumin (BSA)/PBS (Gibco) for 30 min to block nonspecific binding. Then, the cells were incubated in the dark with fluorescein isothiocyanate‐labeled anti‐CD73 (561254, BD Biosciences, USA), anti‐CD90 (561969, BD Biosciences), anti‐CD105 (561443, BD Biosciences), anti‐CD14 (561712, BD Biosciences), anti‐CD34 (560942, BD Biosciences), anti‐CD45 (560976, BD Biosciences, USA), and anti‐HLA‐DR (560944, BD Biosciences, USA) at 4°C for 30 min. Then, the cells were washed twice with 1% BSA/PBS and resuspended in 200 μL of 1% BSA/PBS. Finally, the analysis was performed using a BD FACSCalibur flow cytometer.

### 2.3 Human UC‐MSCs differentiation assays

Adipogenic and osteogenic differentiation assays at the fourth passage were conducted to detect the multilineage differentiation potential of the isolated human UC‐MSCs. UC‐MSCs were induced to differentiate using an Adipogenesis Differentiation Kit (A1007001, Gibco) and Osteogenesis Differentiation Kit (A1007201, Gibco). Lipid accumulation was detected at 7–14 days after induction culture by Oil Red O staining. For osteogenic differentiation, the calcium deposition was detected at 21–28 days after induction culture by Alizarin Red S staining.

### 2.4 Human UC‐MSCs culture on the 3D collagen scaffolds

3D collagen scaffolds were prepared from bovine skin tissue as described previously.^24^ Scaffolds were rinsed with culture medium and placed on 48‐well culture plates. After soaking overnight at 37°C, excess fluid was removed from the scaffolds using a sterile cotton swab. Then, 50 μL of a UC‐MSCs suspension (5 × 10^5^ cells/cm^2^ scaffold) was dripped equally onto each scaffold to observe histological and cellular morphology. The cell‐seeded scaffolds were incubated for 1 h at 37°C in a humidified atmosphere with 5% CO_2_ and then maintained in a complete culture medium for the following experiments. UC‐MSCs‐seeded scaffolds were collected after 3, 12, 24, and 48 h of culture. Some samples were fixed in 4% paraformaldehyde overnight, dehydrated in graded alcohol solutions, and embedded in paraffin. Five millimeter‐thick sections were stained using a standard hematoxylin and eosin (HE) staining protocol for histology. Other samples were fixed in 4% glutaraldehyde and then dehydrated in graded ethanol solutions. After the samples were coated with gold in a JEC‐3000PC (Jeol Inc., Japan), they were observed under a scanning electron microscope (Hitachi S‐3400N II, Tokyo, Japan) for cellular morphology.

UC‐MSCs cell‐seeded scaffold was collected after 1, 3, 5, and 12 h cultured, and the samples were fixed in 4% paraformaldehyde overnight, dehydrated in graded alcohols, and embedded in paraffin. Immunohistochemistry was used to analyze the cell apoptosis and proliferation on collagen scaffold using anti‐Caspase‐3 (ab49822, Abcam, Shanghai, China; 1:250) and anti‐Ki67 antibody (zm0166, ZSGB‐BIO, Beijing, China; 1:200). Immunohistochemistry was performed on 5 μm. The number of Caspase‐3 positive cells and Ki67 positive cells was evaluated using the software of Image‐Pro plus 6.0.

### 2.5 RNA sequencing of UC‐MSCs cultured on collagen scaffolds

To investigate the potential effects of collagen/UC‐MSCs and UC‐MSCs on lung regeneration, cells cultured on dishes and scaffolds were collected after 24 h for mRNA sequencing (RNA‐Seq) by Majorbio Company (Shanghai, China). The mRNA‐seq library was prepared for sequencing using a standard Illumina Truseq™ RNA Sample Prep Kit on the Illumina Hiseq platform, according to the manufacturer's protocols. Bioinformatics analysis was performed using the Majorbio online platform.

### 2.6 Enzyme‐linked immunosorbent assay

HGF and VEGF concentrations in the culture supernatant after cell‐seeded scaffolds were cultured for 48 h were measured by enzyme‐linked immunosorbent assays (ELISAs). The samples were centrifuged at 2500 rpm for 10 min at 4°C to remove cellular debris and then stored at −80°C until further analysis. The supernatants were assayed by an HGF ELISA kit (EK0369, Bosterbio, USA) and a VEGF ELISA kit (EK0539, Bosterbio), according to the manufacturer's instructions.

### 2.7 Rat lung injury model and scaffold/UC‐MSCs transplantation

Animal experiments were performed in accordance with the Chinese Ministry of Public Health (CMPH) Guide and the US National Institute of Health Guide for the care and use of laboratory animals and were approved by the Institute of Genetics and Developmental Biology at the Chinese Academy of Sciences (IGDB‐2018‐IRB‐007). A total of 80 male Sprague–Dawley rats (250–280 g) were housed in a temperature and humidity‐controlled animal room with a 12 h light/dark cycle. All animal studies that meet ARRIVE guidelines were randomly divided into three groups with different treatments to observe the scaffold region at 7, 30, and 60 days post‐operation. In the sham control group, the incision was made to expose the middle lobe of the right lung in the fifth intercostal space, but there was no injured site made in the lung. In the blank 3D collagen group, the excised lung was replaced with a blank collagen scaffold loaded with 100 μL phosphate buffer. In the 3D collagen scaffold/UC‐MSCs group, the excised lung was replaced with a collagen scaffold seeded with 100 μL of a UC‐MSCs suspension (1 × 10^6^ cells/cm^2^ scaffold). At the indicated time points, the rats were sacrificed by acute blood loss. Random numbers were generated using the standard = RANDBETWEEN(1,80) function in Microsoft Excel.

The surgical procedures for the lung injury model are described in our previous study.^24^ Following intraperitoneal anesthesia with chloral hydrate (40 mg/kg body weight), skin preparation, and noninvasive mechanical ventilation, a 2‐cm incision was made in the anterior chest to expose the middle lobe of the right lung in the fifth intercostal space. Then, lung tissue with a volume of 6 × 3 × 2 mm was removed, and the same volume of a 3D collagen scaffold seeded with UC‐MSCs was implanted into the lung tissue gap. Then, the surgery site was irrigated with normal saline, and the musculature and skin were closed in separate layers with sutures. Finally, the rats were returned to their cages and kept warm to promote recovery from anesthetization.

The details of the UC‐MSCs seeded operating procedure are as follows: (1) The isolated fresh UC‐MSCs were passaged about 2–5 generations and used for morphological observation, flow cytometry analysis, differentiation ability detection, and karyotype analysis for cell identification. The obtained high‐quality UC‐MSCs were partially frozen in liquid nitrogen for reserve at the concentration of 1 × 10^6^ cells/mL (frozen storage solution: 90% fetal bovine serum and 10% dimethylsulfoxide).^38^ (2) Another part of the fresh, high‐quality UC‐MSCs were seeded in a complete medium for amplification culture. (3) Sterilized 3D collagen scaffolds with a volume of 6 × 3 × 2 mm were rinsed with culture medium and soaked overnight at 37°C, excess fluid was removed and then transferred to a new 6‐well plate. (4) Hundred microliter of a UC‐MSCs suspension (1 × 10^6^ cells/cm^2^ scaffold) was dripped equally onto each scaffold and cultured at 37°C for 3 h until the cells adhered to the scaffolds wall. (5) PBS was used to thoroughly remove the medium on the scaffold, and the number of cells in the washing solution was observed under a light microscope to confirm that UC‐MSCs were not washed away. (6) Biomimetic scaffold‐based stem cell implantation was transplanted into the lung tissue‐injured gap and fixed using two 8/0 monofilament nylon interrupted sutures at each side of the anastomosis.

### 2.8 Long‐term labeled human UC‐MSCs in vitro and in vivo

CM‐Dil (C7000, Molecular Probes, Invitrogen, USA) was used to label UC‐MSCs before scaffold/UC‐MSCs implantation to track the stem cells, according to the manufacturer's instructions. The labeling efficiency in vitro was detected from passages three to seven during cell culture. After scaffold/CM‐Dil‐labeled UC‐MSCs implantation, lung tissues containing the scaffold/UC‐MSCs complex were collected at days 7, 30, and 60, embedded in optimum cutting temperature medium, sectioned at 6 μm thicknesses, stained with 4′,6‐diamidino‐2‐phenylindole for 5 min, and observed under a fluorescence microscope. The CM‐Dil^+^ signal was evaluated by Image‐Pro Plus 6.0 software.

### 2.9 Histological analysis

Rats were anesthetized by an intraperitoneal injection of chloral hydrate (40 mg/kg body weight) at 7, 30, and 60 days post‐surgery. The operative region of lung tissue was collected and fixed in 4% paraformaldehyde overnight, dehydrated in graded alcohol solutions, and embedded in paraffin. Sections of 5 μm in thickness were prepared transversally and stained using standard hematoxylin‐eosin and Masson's trichrome staining protocols.

### 2.10 Immunofluorescence analysis

Rats' lung tissues containing collagen/UC‐MSCs complexes were collected at 7, 30, and 60 days post‐surgery, embedded in optimum cutting temperature medium, and sectioned at 6 μm thicknesses. The sections were stained with the following antibodies: anti‐CD31 (ab28364, Abcam; 1:50), anti‐SPC (ab211326, Abcam; 1:500), anti‐AQP5 (ab92320, Abcam; 1:200), anti‐CD31 (ab24590, Abcam; 1:50), and anti‐Ki67 (zm0166, ZSGB‐BIO, Beijing, China; 1:200). The number of positive cells was evaluated by Image‐Pro Plus 6.0 software.

### 2.11 Micro‐CT testing

Rats were anesthetized with 10% chloral hydrate and placed in the chamber of a computed tomography (CT) scanner for small animals (Quantum GX; PerkinElmer, Massachusetts, USA). CT scanning was performed and image acquisition was conducted under respiratory gating. The total lung volume was calculated using Analyze 11.0 software.

### 2.12 Statistical analysis

Data were presented as mean ± standard deviation (SD). Normality tests were performed before comparison between groups. Differences between groups were compared using a two‐tailed Student's *t*‐test or ANOVA. Statistics were calculated with Graph Pad Prism 6 software. Differences were considered significant as *p < 0.05 and **p < 0.01.

## 3 RESULTS

### 3.1 Characterization of collagen/UC‐MSCs scaffolds

**FIGURE 1:**
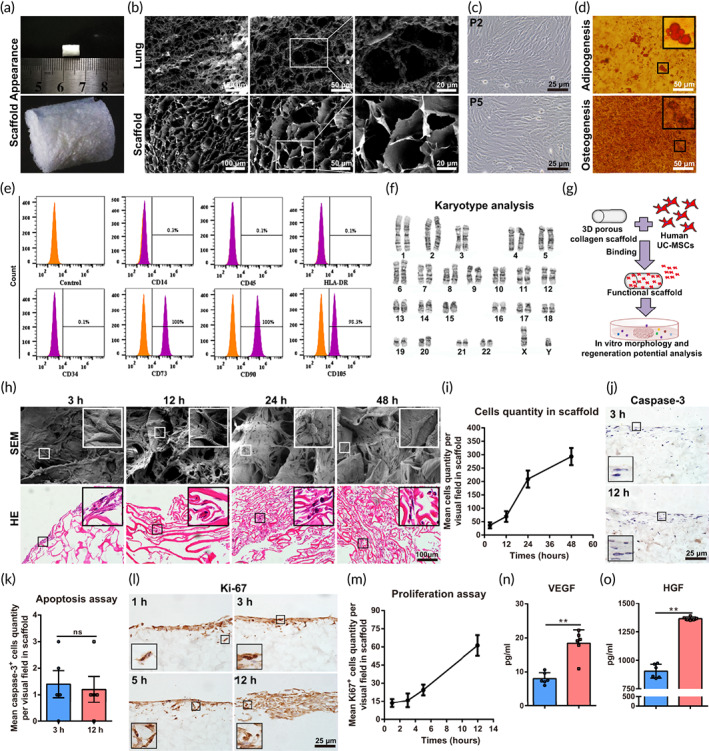
Macrograph and characterization of an artificial 3D biomimetic scaffold‐based mesenchymal stem cells (MSCs) implant in vitro. (a) Columnar and porous structure of 3D collagen scaffold with a 0.5 cm diameter and 0.7 cm height. (b) SEM images showing biomimetic structures of the scaffold and lung section. (c) Homogenous, spindle‐like morphology of human umbilical cord (UC) MSCs at the second and fifth passages. (d) The multilineage differentiation potential of human UC‐MSCs, adipogenesis was detected by Oil red O staining to show intracellular lipid accumulation and osteogenesis was observed by Alizarin Red S staining to show calcium deposition. (e) Identification of human UC‐MSCs phenotype by flow cytometry. (f) A representative chromosome spread of normal diploid UC‐MSCs with 22 pairs of autosomes and 1paris sex chromosomes. (g) Schematic diagram of the biocompatibility experiment and lung regeneration potential analysis of UC‐MSCs and scaffolds in vitro. (h) SEM images of the cellular morphology of UC‐MSCs and HE‐stained images of scaffold longitudinal sections at 3, 12, 24, and 48 h of culture on the collagen scaffold. (i) The curve of UC‐MSCs quantity inside the scaffolds. (j) Immunohistochemical staining was used to evaluate the apoptosis of UC‐MSCs on the collagen membrane with an anti‐Caspase‐3 antibody at 3 and 12 h after cell seeding. (k) Apoptosis assay of UC‐MSCs on collagen by counting the number of Caspase‐3‐positive cells. (l) Immunohistochemical staining was used to evaluate the proliferation of UC‐MSCs on the collagen membrane with an anti‐Ki67 antibody at 1, 3, 5, and 12 h after cell seeding. (m) Proliferation curve of UC‐MSCs on collagen by counting the number of Ki67‐positive cells. Concentrations of vascular endothelial growth factor (VEGF) (n), and hepatocyte growth factor (HGF) (o) in the culture supernatants of collagen/UC‐MSCs constructs were measured by ELISAs. Data represent the mean ± SD, *n* = 5, **p < 0.01.

As shown in Figure 1a, a collagen scaffold is the porous structure of 0.5 cm in diameter and 0.7 cm in height which had similar structural characteristics as the normal lung (Figure 1b).^24^ UC‐MSCs isolated from fresh human umbilical cord tissues showed spindle‐shaped adherent growth, and until the fifth passage, the cells still maintained normal cell morphology and exhibited multilineage differentiation potentials for adipocytes and osteoblasts (Figure 1c,d), which were all positive for CD73, CD90, and CD105 and negative for CD14, CD45, CD34, and HLA‐DR (Figure 1e), indicated the nice purity. The safety of UC‐MSCs was also evaluated prior to the experiment. The human MSCs have normal karyotypes without mutation, and the cell culture survival rate can reach 98% (Figure 1f). UC‐MSCs attachment survival, proliferation, apoptosis, and migration were used to evaluate cell responses on the scaffold (Figure 1g). Collagen scaffolds were cut into small pieces. After sterilization, cleaning, soaking, and removing excess fluid on the surface, the collagen scaffold was seeded with cells and cultured for 2 h. UC‐MSCs cultured in the 3D collagen scaffold were collected at 3, 12, 24, and 48 h for histomorphology and cell morphology analysis of longitudinal sections. The results showed that UC‐MSCs could adhere, survive (Figure 1h), proliferate (Figure 1l,m) on the scaffold, and migrate along the pores to the inside of the scaffold (Figure 1i) without apoptosis (Figure 1j,k), suggesting excellent biocompatibility of the collagen scaffold.

Paracrine signaling is one of the major mechanisms of MSC‐mediated lung regeneration. The emergence of new capillaries and the mobilization of alveolar type II epithelial cells (AT II cells) are important steps in alveolar‐like structure formation and functional recovery, which are closely related to the driving of VEGF and HGF.^39^, ^40^, ^41^, ^42^, ^43^, ^44^ High levels of VEGF and HGF (Figure 1j,k) were detected in the culture supernatants of the collagen/UC‐MSCs group by ELISAs, which might be beneficial for the survival and migration of endothelial and alveolar epithelial cells after scaffolds transplantation.^45^, ^46^, ^47^ The concentration of VEGF in the supernatants of collagen/UC‐MSCs (18.42 ± 1.61 pg/mL) was higher than that in the MSC group (8.07 ± 0.71 pg/mL; *n* = 5, *p* < 0.01). Moreover, the concentration of HGF in the supernatants of collagen/UC‐MSCs (1365 ± 4.291 pg/mL) was higher than that in the MSC group (914.3 ± 10.28 pg/mL; *n* = 5, *p* < 0.01).

**FIGURE 2:**
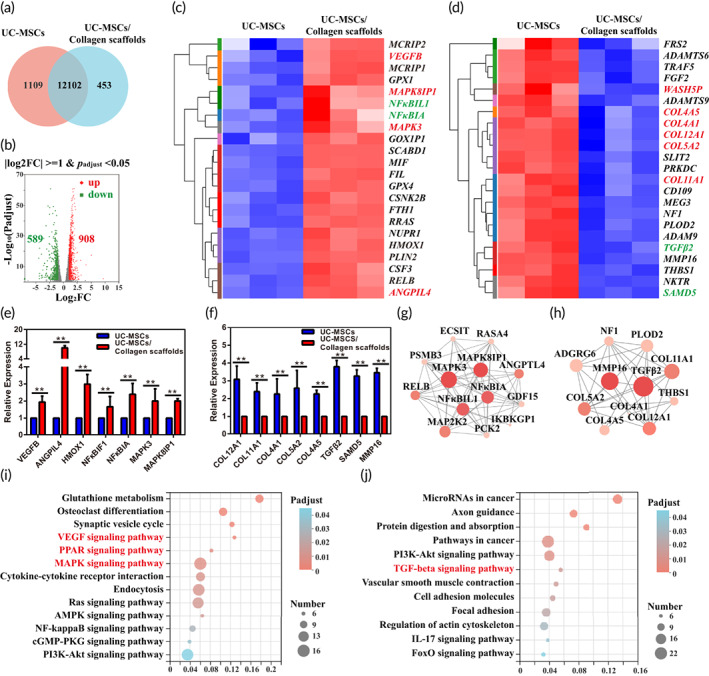
Therapeutic potential analysis of umbilical cord mesenchymal‐derived stem cells (UC‐MSCs)/collagen scaffolds in situ lung regeneration. (a) Venn diagram showing the intersection gene between UC‐MSCs and UC‐MSCs/Collagen scaffolds groups. (b) Volcano plot of differential gene expression in the UC‐MSCs/Collagen scaffolds group compared with the UC‐MSCs group. Heat map of (c) upregulated genes and (d) downregulated genes involved in the lung regeneration microenvironment of UC‐MSCs/Collagen scaffolds compared with UC‐MSCs group (fold change ≥2 and *p*‐value <0.05). Quantitative analysis of (e) upregulated gene expression related to anti‐inflammation, anti‐apoptosis, and tissue regeneration and (f) downregulated gene expression involved in fibrosis. The protein‐protein interaction (PPI) network of (g) upregulated and (h) downregulated genes, respectively, is involved in angiogenesis, proliferation, migration and anti‐inflammation, and anti‐fibrosis. KEGG pathway enrichment analysis of differentially expressed genes (DEGs). (i) Thirteen significantly enriched KEGG pathways of upregulated and (j) twelve significantly enriched KEGG pathways of downregulated DEGs. Data represent the mean ± SD, *n* = 5, **p < 0.01.

To further clarify the therapeutic potential of the collagen/UC‐MSCs scaffolds in situ lung regeneration, cells cultured on the collagen scaffold were collected after 24 h cultured for RNA‐seq analysis (Bioproject Accession: PRJNA580461), which was in the period of stable proliferation and migration. Cell cultured in the cell‐culture dish were taken as controls. The results showed that 12,102 genes were shared, 453 genes were unique to the UC‐MSCs group and 1109 genes were unique to the control group according to the Venn diagram (Figure 2a). Volcano plots showed 1497 significant differentially expressed genes (DEGs), with 908 upregulated genes and 589 downregulated genes according to the cut‐off criteria (>2‐fold, *p* < 0.05; Figure 2b).

The effect of collagen/UC‐MSCs scaffolds on the expression of angiogenesis, proliferation, and migration genes related to lung regeneration, as well as inflammation and fibrosis genes related to lung recovery was investigated. The difference in gene expression between the UC‐MSCs and collagen/UC‐MSCs scaffolds groups was presented in the heatmap (Figure 2c,d). The expression of angiogenesis genes (*VEGFB, ANGPIL4*), anti‐inflammation genes (*HMOX1, PLIN2*),^48^, ^49^ negative regulatory genes of apoptosis (*NFκBIA, NFκBIF1*) and survival and proliferation genes (*MAPK3, MAPK8IP1*) was upregulated (Figure 2E) and suggested an obvious regeneration potential. The expression of several important fibrosis genes (*COL12A1, COL11A1, COL4A1, COL5A2, COL4A5, TGFβ2, SAMD5, MMP16*) was also significantly downregulated (Figure 2f), indicated the potential that collagen/UC‐MSCs scaffolds relieved the fibrosis which might be induced by collagen scaffold transplantation. The protein‐protein interaction network analysis was also performed to identify the changes in the expression of lung regeneration and recovery genes (Figure 2g,h). The results confirmed the probable leading role of MAPK in regeneration and TGFβ in anti‐fibrosis. Upregulation of other related proteins suggested collagen/UC‐MSCs inhibit inflammation and apoptosis, and downregulation of other proteins indicated the potential of collagen/UC‐MSCs to reduce collagen deposition. As shown in Figure 2i, the Kyoto Encyclopedia of Genes and Genomes (KEGG) pathway enrichment analysis, the DEGs in the collagen/UC‐MSCs group were focused on the VEGF and MAPK signaling pathways associated with the tissue regeneration mechanisms. Furthermore, the KEGG analysis indicated that the key enrichment signaling pathways such as PPAR and IκB signaling pathways, were also centered on relieving inflammation and apoptosis. Notably, the fibrosis‐associated signaling pathways, TGFβ signaling pathways, were significantly inhibited after UC‐MSCs were loaded (Figure 2j).

In brief, we constructed an artificial 3D biomimetic scaffold‐based MSCs implant which had good biocompatibility and paracrine capacity. It may have a great potential for lung regeneration with the potential to promote endothelial and epithelial cell survival, proliferation, and migration, as well as downregulate inflammation, apoptosis, and fibrosis.

### 3.2 UC‐MSCs migration and long‐term survival and proliferation and injured lungs

**FIGURE 3:**
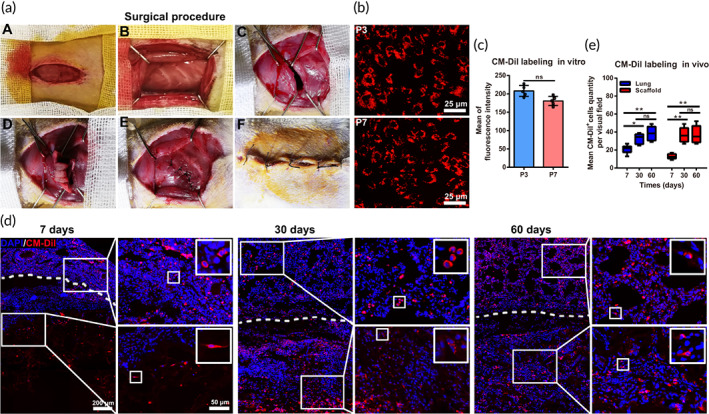
Establishment of a model of lung partial resection in rat and tracking of long‐term labeled human umbilical cord mesenchymal‐derived stem cells (UC‐MSCs) in vivo. (a) (A–F) Surgical procedure of model establishment. (A) An incision was made in the anterior chest; (B) The fifth intercostal space of the right margin of the sternum was exposed; (C) Around an 8 mm wedge incision was made; (D) The middle lobe of the right lung was partially resected, and a 3D collagen scaffold was implanted; (E) The intercostal space was sutured; (F) The skin incision was sutured. (b) UC‐MSCs were labeled with CM‐Dil from passage three to seven in vitro. (c) Statistical analysis of CM‐Dil‐labeled UC‐MSCs at passage three and seven. Data represent the mean ± SD, *n* = 5, ns: no statistical significance. (d) Tracking of implanted UC‐MSCs in the operative lung and scaffold at 7, 30, and 60 days post‐surgery. Lung tissue is above the dotted white line, and the scaffold is below. The image on the right is the higher magnification of the small white boxed area of the image on the left. The large white boxes of the images on the right indicate higher magnification images of the small boxed area. (e) Statistical analysis of CM‐Dil‐labeled UC‐MSCs in vivo. Data represent the mean ± SD, *n* = 5, ns: no statistical significance, **p < 0.01.

CM‐Dil, a long‐term cellular labeling dye, was used to track UC‐MSCs. As shown in Figure 3b,c, up to the seventh passage of UC‐MSCs, the fluorescence intensity was detected, and there was still good labeling efficiency in vitro. Next, a model of right lung partial resection of the middle lobe was established in rats. The rats had a stable survival rate for further investigation of lung regeneration (Figure 3a). Then, collagen loaded with CM‐Dil‐labeled UC‐MSCs was implanted into the injury site to track UC‐MSCs in vivo, and samples were collected at 7, 30, and 60 days. Immunofluorescence showed that the transplanted UC‐MSCs survived in the scaffold in vivo. At 7 days post‐implantation, some UC‐MSCs had migrated to the injured lung tissue from the scaffold, while others remained on the scaffold and maintained adherent growth (Figure 3d). At 30 days post‐implantation, the count of CM‐Dil^+^ cells was significantly increased compared to it at 7 days post‐implantation in the lung and scaffold (Figure 3e), indicating that the implanted UC‐MSCs had a proliferation ability in vivo. The CM‐Dil^+^ signal was still observed in the scaffold and lung tissue even at 60 days post‐implantation (Figure 3d), and there was no significant difference in the number of CM‐Dil^+^ cells on either the scaffold or the residual lung between 30 and 60 days post‐implantation (Figure 3e), demonstrated that the UC‐MSCs had a long‐term survival and proliferation ability in the lung and scaffold.

### 3.3 Histological changes in scaffolds after the implantation of the collagen/UC‐MSCs scaffold

**FIGURE 4:**
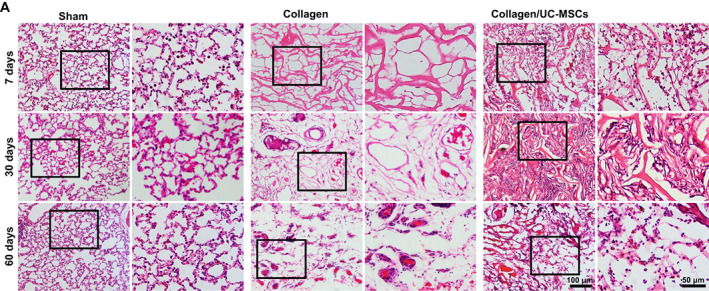
Histologic morphology of scaffolds in rats. Histological observation of collagen scaffolds by hematoxylin and eosin staining at 7, 30, and 60 days post‐surgery. The high‐power image next to is the higher magnification of the small black boxed area in scaffold.

Next, we observed the histological morphology of the scaffold area by HE staining at 7, 30, and 60 days post‐surgery. As shown in Figure 4, there were few cells in the scaffold of the collagen group, whereas some tissue‐like structures had formed in the scaffold of the collagen/UC‐MSCs group at 7 days post‐surgery. At 30 days post‐surgery, more regenerated tissue‐like structures grew along the scaffold in the collagen/UC‐MSCs group, and more cells had migrated into the scaffold of the collagen group, but the number of cells was still small and the cells were scattered. Interestingly, at 60 days post‐surgery, vascular‐like structures in scaffolds were observed in both collagen and collagen/UC‐MSCs groups. There were still relatively few cells in the collagen group, but alveolus‐like structures were clearly observed in the collagen/UC‐MSCs group. These results suggested that both collagen and collagen/UC‐MSCs induced vascularization, and that collagen/UC‐MSCs directed the formation of alveolus‐like structures.

### 3.4 Collagen/UC‐MSCs scaffolds inhibit post‐operative lung inflammation and fibrosis

**FIGURE 5:**
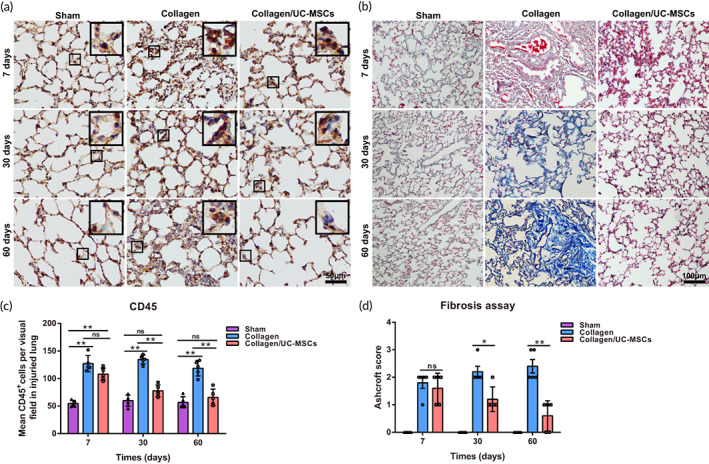
Histological evaluation of inflammation and fibrosis in injured lungs. (a) Injured lung was stained with an anti‐CD45 antibody at 7, 30, and 60 days post‐surgery in the three groups. (b) Histological observation of injured lung by Masson's trichrome staining at 7, 30, and 60 days post‐surgery. (c) Quantification of CD45‐positive cells in residual lungs at 7, 30, and 60 days post‐surgery. (d) Fibrosis assay based on Masson's trichrome‐stained images, according to the Ashcroft score. Data represent the mean ± SD, *n* = 5, *p < 0.05, **p < 0.01, ns: no statistical significance.

Inflammation and fibrosis are major obstacles in the process of lung recovery and regeneration. Inflammation was assessed by CD45 staining of the injured region (Figure 5a). Statistical analysis of the number of CD45‐positive cells (Figure 5c) showed that, although collagen might induce mild inflammation at the early stage post‐surgery, the inflammation was decreased gradually over time by UC‐MSCs. Fibrosis of injured lungs was evaluated by Masson's trichrome staining (Figure 5b) and the modified Ashcroft score (Figure 5d). Collagen and the surgery‐induced mild fibrosis, whereas UC‐MSCs exerted an anti‐fibrosis effect and reduced fibrosis. Thus, collagen/UC‐MSCs inhibited post‐operative lung inflammation and fibrosis and provided a good regenerative environment.

### 3.5 Vascularization on collagen/UC‐MSCs scaffolds

**FIGURE 6:**
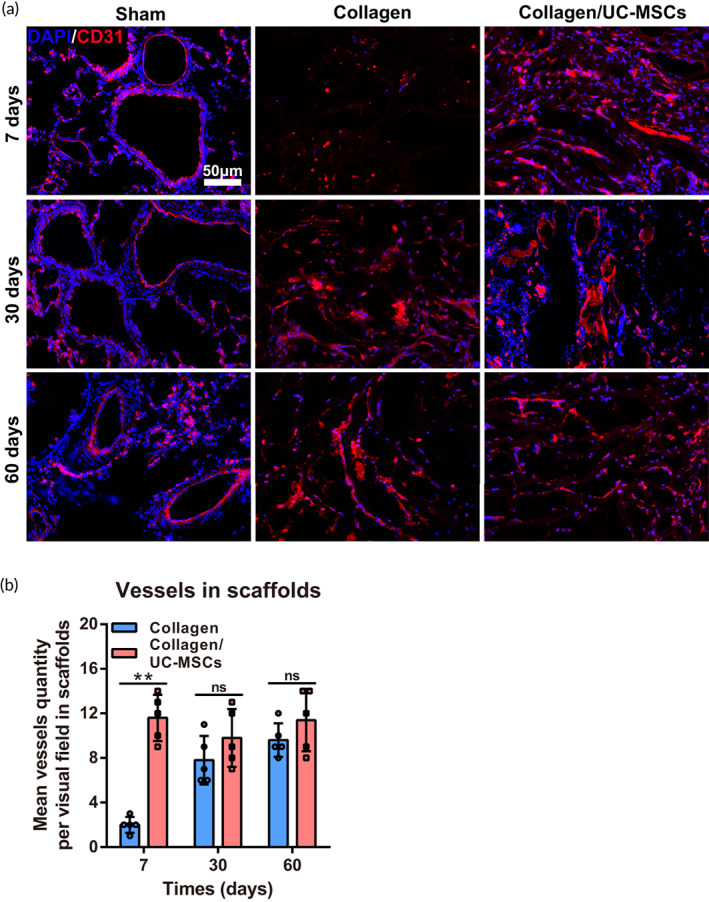
Effect of collagen/umbilical cord mesenchymal‐derived stem cells (UC‐MSCs) on angiogenesis in scaffolds. (a) Immunofluorescence staining of CD31 was used to assess angiogenesis in scaffolds. (b) Quantification of vessels in scaffolds at 7, 30, and 60 days post‐surgery. Data represent the mean ± SD, *n* = 5, **p < 0.01, ns: no statistical significance.

Alveolar capillaries exist between adjacent alveolar walls and fill the alveolar septum, are an essential part of lung tissue for gas exchange. The emergence of new capillaries is an important step in alveolar formation and functional recovery.^40^ Next, we investigated angiogenesis by CD31 immunofluorescence staining in scaffolds after implantation (Figure 6a). At 7 days post‐surgery, the number of CD31‐positive cells in scaffolds of the collagen/UC‐MSCs group was obviously higher than that in the collagen group, and some microvascular had formed in scaffolds (11.66 ± 1.25; *n* = 5, *p* < 0.01). At 30 days post‐surgery, endothelial cells had also migrated into the scaffolds of the collagen group and formed vessels, and the number of vessels was no significantly different than that in the collagen/UC‐MSCs group (Figure 6b), but the vessels in the collagen/UC‐MSCs were more mature. These results indicated that collagen directed the migration of endothelial cells to form vessels, and collagen/UC‐MSCs accelerated the vascularization process of scaffolds.

### 3.6 AT II and AT I cell behavior on collagen/UC‐MSCs scaffolds

**FIGURE 7:**
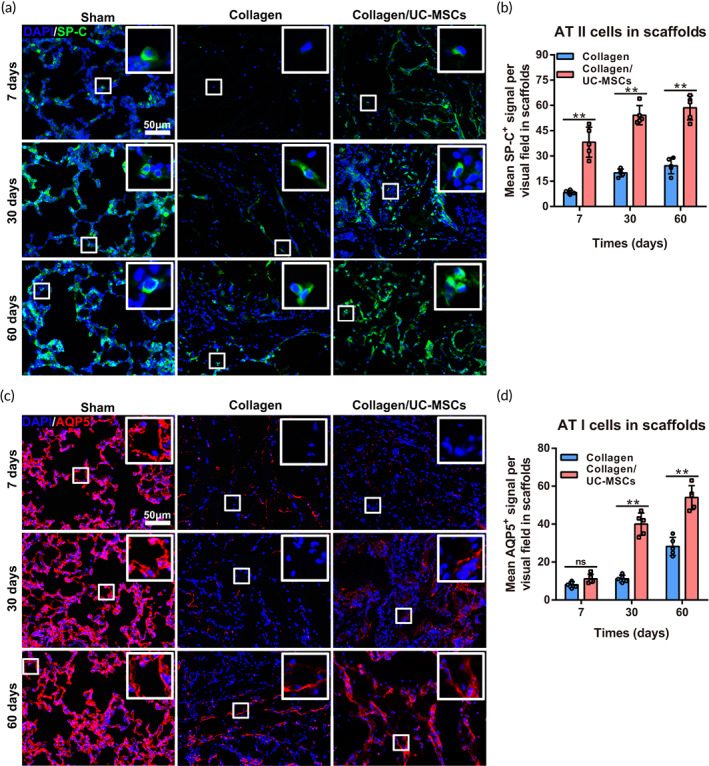
Effect of collagen/umbilical cord mesenchymal‐derived stem cells (UC‐MSCs) on alveolar type (AT) II and AT I cells behavior in scaffolds. (a) Immunofluorescence staining of SP‐C was performed to identify AT II cells in scaffolds. (b) Quantification of SP‐C‐positive cells in scaffolds at 7, 30, and 60 days post‐surgery. (c) Immunofluorescence staining of AQP5 was used to detect AT I cells in scaffolds. (d) Quantification of AQP5‐positive signal in scaffolds at 7, 30, and 60 days post‐surgery. Data represent the mean ± SD, *n* = 5, **p < 0.01, ns, no statistical significance.

As the basic unit of gas exchange, alveoli are mainly composed of squamous AT I cells and cuboidal, surfactant‐secreting AT II cells. AT I cells are highly differentiated cells that mainly mediate gas exchange and are unable to self‐renew and migrate. AT II cells secrete surfactant that prevents alveolar collapse and act as stem/progenitor cells after AT I cell injury, which renews AT I cells and produces slowly expanding clonal foci of alveolar renewal.^32^ Therefore, the behavior of AT II cells (Figure 7a,b) and AT I cells (Figure 7c,d) was assessed after scaffold implantation. At 7 days post‐surgery, there were many AT II cells in scaffolds of the collagen/UC‐MSCs group (38.51 ± 8.87), whereas almost no AT II cells were found in scaffolds of the collagen group (8.5 ± 1.11; *n* = 5, *p* < 0.01). As expected, few AT I cells were observed in scaffolds of both collagen (8 ± 1.63) and collagen/UC‐MSCs (11.3 ± 2.13) groups (*n* = 5, *p* < 0.01). At 30 days post‐surgery, the number of AT II cells in scaffolds of the collagen group (19.7 ± 2.16) was increased but still significantly lower than that in the collagen/UC‐MSCs group (55 ± 5.38; *n* = 5, *p* < 0.01) that maintained a stable level. Encouragingly, more AT I cells were observed in scaffolds of the collagen/UC‐MSCs group (40.35 ± 3.56), and a few AT I cells were present in scaffolds of the collagen group (11.21 ± 2.16; *n* = 5, *p* < 0.01). At 60 days post‐surgery, the number of AT II cells and AT I cells in the scaffolds of the collagen group was still significantly lower than that in the collagen/UC‐MSCs group. These results supported the abovementioned classical view and demonstrated that collagen scaffolds were conducive for the migration and survival of AT II cells and renewed AT I cells, and collagen/UC‐MSCs enhanced these processes.

### 3.7 General morphology recovery of the injured lung and alveolar structures on collagen/UC‐MSCs scaffolds

**FIGURE 8:**
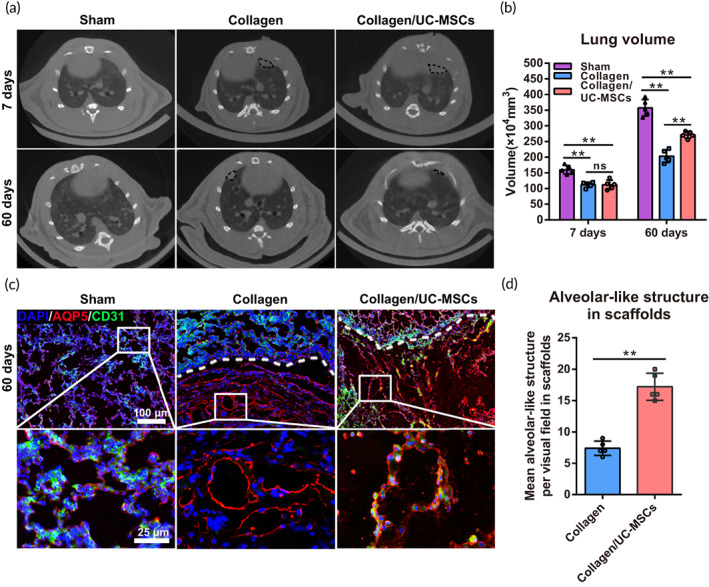
General morphological recovery and alveolus‐like structure formation in the injured lung. (a) Micro‐computed tomography (CT) scanning showed the general morphology of the lung operative region at 7 and 60 days post‐surgery in sham, control, and collagen/umbilical cord mesenchymal‐derived stem cells (UC‐MSCs) groups. Defected tissue and the operative area are highlighted with a dotted black line. (b) Quantification of lung volumes by micro‐CT scanning at 7 and 60 days post‐surgery in the three groups. (c) Double immunofluorescence staining of AQP5 (red) and CD31 (green) in scaffolds at 60 days post‐surgery. Higher magnification images of areas indicated by white boxes are shown. (d) Functional quantification of alveolus‐like structures consisting of CD31‐ and AQP5‐positive cells in scaffolds at 60 days post‐surgery. Data represent the mean ± SD, *n* = 5, **p < 0.01; ns, no statistical significance.

Finally, functional recovery of the injured lung was evaluated by micro‐CT scanning that was used to assess the lung's general morphology (Figure 8a) and calculate the lung volume (Figure 8b). The results showed that, although the general morphology of the injured lung was restored at 60 days post‐surgery, the surgical gap still existed in the collagen group, whereas the lung morphology was obviously restored in the collagen/UC‐MSCs group. The lung volume of the collagen group was also significantly lower than that in the collagen/UC‐MSCs group at 60 days post‐surgery.

The functional unit for gas exchange of alveoli mainly consists of AT I cells and microvascular. Therefore, double immunofluorescence of CD31 and AQP5 was used to determine the status of the new functional units in the scaffolds (Figure 8c). At 60 days post‐surgery, only a few alveolus‐like structures were observed at the junction of the lung and scaffold in the collagen group (7.41 ± 0.51). However, significantly more alveolus‐like structures were formed in scaffolds of the collagen/UC‐MSCs group (17.21 ± 0.97; *n* = 5, *p* < 0.01). These results confirmed that collagen‐guided endogenous regeneration of the injured lung, and collagen/UC‐MSCs might provide a stable regenerative niche that promotes efficient and orderly regeneration of alveoli.

## 4 DISCUSSION

In the present study, we developed a new strategy to fabricate an artificial niche of biomimetic scaffold‐based implants and demonstrated that the biomimetic collagen scaffolds directed alveolar and microvascular regeneration as well as lung recovery after severe lung injury, and UC‐MSCs facilitated this process. Vascularization and many alveolus‐like structures were observed in the residual scaffolds. These findings might be closely related to the migration and proliferation of endogenous progenitor/stem cells in the injured lung tissue and scaffold under the stimulus of the artificial regeneration niche.

Lung regeneration remains a major challenge because of the poorly defined stem cell population and its complex and unclear relationship with stem/progenitor cells at various anatomical locations along the proximal–distal axis of the airway.^3^ For the most acute and lethal lung injury, lung transplantation remains as the only solution other than the mitigating treatments available, but donors and side effects severely limit its application and efficacy.^1^ Hence, artificial lung scaffolds are emerging as a potential substitute,^29^ but still have limitations such as inflammation, edema, intravascular coagulation, and failure to establish the endothelial barrier function in vivo.^50^ Stem/progenitor cell‐based regenerative therapy may be another strategy for such injury. Among the various cells with clinical potential, induced pluripotent stem cells have been successfully differentiated into alveolar and airway lineages in vitro, but the tumorigenic risk remains to be evaluated in vivo.^51^, ^52^ The differentiation potential and regulatory mechanism have not been determined for tissue‐resident progenitor/stem cells isolated from lungs.^2^ Therefore, mesenchymal stem cells (MSCs) remain a reliable strategy owing to their widely recognized paracrine and immunomodulatory mechanisms, and easy isolation and handling.^23^ However, the transplantation efficiency of UC‐MSCs is low because few cells are retained and survive in the lung, which limits their application.

The stem cell niche is an anatomical microenvironment structure consisting of both cellular and acellular elements which control the behavior of stem cells and the balance between self‐renewal and differentiation to maintain tissue homeostasis of the organism.^53^, ^54^ In a previous study, MSCs supported epithelial integrity during homeostasis and promoted epithelial regeneration after lung injury, which were attributed to the cellular element of stem cell niche.^55^ An acellular lung scaffold is the most common artificial lung, which provides an adhesion surface and growth environment for epithelial and endothelial cells, which are essential noncellular components for a stem cell niche. Collagens, especially collagen type I, are the main component of most acellular lung scaffolds and widely used in tissue regeneration.^56^ In our study, we developed a 3D culture system by preparing a 3D porous collagen scaffold loaded with UC‐MSCs and demonstrated that the collagen scaffold was suitable for cell adhesion, nutrient and oxygen delivery, cell survival, proliferation, and even long‐term cell retention, which was confirmed by tracking UC‐MSCs using CM‐Dil labeling in vivo and greatly improved the therapeutic efficiency of UC‐MSCs.

Paracrine signaling, including immunomodulation, is believed to be one of the major mechanisms for UC‐MSCs‐mediated tissue repair. Destruction of the lung alveolus‐capillary membrane barrier is the primary characteristic of acute lung injury.^57^ In previous studies, VEGF^44^ and HGF^25^ alleviated such destruction by antagonizing vascular inflammation and promoting the survival of endothelial cells and angiogenesis. Other studies have also indicated that HGF ameliorates lung injury and restores the integrity and permeability of endothelial cells in emphysema and fibrosis models.^58^, ^59^ In our 3D cultures, the expression levels of VEGF and HGF were significantly increased compared with two‐dimensional (2D)‐cultured cells. In addition, gene expression differences between 2D‐ and 3D‐cultured cells were detected by RNA sequencing, which showed upregulation of lung recovery and regeneration‐related genes and signaling pathways. Thus, an artificial niche was constructed, and the above evidence implied its potential for lung regeneration in vivo.

As expected, in animal experiments, the external artificial niche and endogenous stem cells and niche had good communication. Compared with traditional stem cell delivery, the collagen scaffold retained more stem cells in the injured lung. Some UC‐MSCs migrated from the scaffold to the entire damaged lung at 7 days after implantation. Previous reports^60^, ^61^ showed that UC‐MSCs promoted endothelial cell proliferation, sprouting, and migration, and collagen provided an attachment surface, which induced the formation of vessel‐like structures in the scaffold at 14 days post‐surgery. The early establishment of blood supply strengthened the connection between the scaffold and microenvironments, which was conducive for the recovery and regeneration in the next stage. The UC‐MSCs also protected the integrity of epithelial cells, leading to some AT II cells appearing in scaffolds in the early stage (7 days) of regeneration. With the AT II cell survival and colonization in scaffolds, AT I cells were also found in scaffolds until 30 days post‐surgery, demonstrating the classical view that AT II cells as adult stem/progenitor cells renew AT I cells. In the process of regeneration, the mild inflammation and fibrosis caused by surgery and collagen had little effect owing to the strong paracrine factors of UC‐MSCs exerting anti‐inflammation and anti‐fibrosis effects. Finally, although the general morphological recovery and the number of alveolus‐like structures of the collagen scaffold alone were significantly lower than those in the collagen/UC‐MSCs group, these features demonstrated the effectiveness of collagen.

The xenogeneic immune rejection is the basic problem to be faced in evaluating the efficacy of human UC‐MSCs transplantation using a rat lung resection injury model. But first, human‐derived UC‐MSCs have the properties with low immunogenicity,^62^ and there was no significant immune rejection observed in a large number of preclinical studies, which were mainly in rat injury models.^19^, ^63^, ^64^ Second, UC‐MSCs also have strong immunoregulatory and immunosuppressive effects, which support the application in allogeneic and xenogenic transplantation, and UC‐MSCs are often used to avoid the graft versus host diseases, which occur in recipients after organ transplantation.^65^, ^66^, ^67^, ^68^ Third, from the results of this study (Figure 5A), the UC‐MSCs alleviated the mild inflammation induced by collagen scaffolds transplantation at an early stage post‐surgery. Macrophages are important immune cells in lungs which play a vital role in defense functions including phagocytosis, immunity, and cytokine secretion.^69^ From the perspective of mechanism, it is more convincing that UC‐MSCs improved the regeneration microenvironment by reducing inflammatory cytokine secretion of macrophage, or promoting M2 macrophage polarization and enhanced immunosuppressive factor secretion, which meet the previous studies between MSCs transplantation and endogenous macrophage behaviors in acute lung injury,^70^, ^71^, ^72^, ^73^, ^74^ rather than caused by the xenogenic effects. In addition, macrophages might also regulate the degradation of collagen scaffolds with the formation of alveolar‐like structures.

Despite the findings indicating that the biomimetic scaffold‐based MSC implants exerted excellent lung regeneration effects after acute lung injury, the precise mechanism was unclear. And the artificial implant needs to rely on the presence of endogenous stem/progenitor cells in the residual lung. Therefore, the clinical potential is restricted to partial lung resection and other localized lung diseases. All lung diseases that can cause severe lung destruction and require local lung resection are indications including severe lung trauma from traffic accidents, and construction accidents, and severe lung diseases such as tuberculosis, local idiopathic pulmonary fibrosis, and bronchiectasis. Lung transplant rejection might be the biggest obstacle to the application of the existence of lung allografts. However, due to their immunosuppressive properties, various MSCs are considered potential candidates for the prevention of graft rejection,^75^, ^76^, ^77^, ^78^ and several clinical trials of MSCs for allografts rejection^79^ after lung transplantation have been conducted to evaluate the efficacy and safety for the treatment of lung disease according to clinicaltrials.gov, such as the Phase I study of “the Safety and Feasibility of Mesenchymal Stem Cells to Induce Remission in Lung Transplant Patients Experiencing Treatment‐Refractory Moderate Lung Rejection” (ClinicalTrials.gov Identifier: NCT02181712) carried by Mayo Clinic in Florida, United States, which indicates the feasibility and the application prospect of MSCs. Thus, the prospect of artificial lung regeneration niche remains considerable.

## 5 CONCLUSION

Lung regeneration after partial injury is closely related to the endogenous stem/progenitor cells, which depends on the existence of normal lung tissue and a beneficial regeneration microenvironment. In this work, we successfully constructed an artificial lung regeneration niche consisting of a 3D biomimetic collagen scaffold and human UC‐MSCs, which provides an effective communication with residual lung to promote lung regeneration. First, UC‐MSCs loaded on collagen scaffolds exerted strong paracrine effects and some UC‐MSCs migrated to the lung from scaffolds and had long‐term retention to suppress inflammation and fibrosis in residual lungs, and promoted vascular endothelial cells and alveolar stem cells (AT II cells) to enter the scaffolds. Then, under the guidance of collagen scaffolds and the stimulation of the remaining UC‐MSCs, vascular and alveolar‐like structures were formed in scaffolds region. The artificial 3D biomimetic scaffold‐based MSCs implants induced in situ lung regeneration and recovery after lung destruction, provide a promising direction for tissue engineering and stem cell strategies in lung regeneration and have a great clinical potential for patients with severe lung damage.

## AUTHOR CONTRIBUTIONS

**Linjie Wang:** Formal analysis (lead); investigation (lead); methodology (equal); visualization (lead); writing – original draft (lead). **Meng Feng:** Investigation (equal); methodology (equal). **Yazhen Zhao:** Project administration (supporting). **Bing Chen:** Project administration (lead). **Yannan Zhao:** Conceptualization (equal); funding acquisition (equal); writing – review and editing (equal). **Jianwu Dai:** Conceptualization (lead); funding acquisition (lead); writing – review and editing (equal).

## CONFLICT OF INTEREST STATEMENT

The authors declare that they have no competing interests.

### PEER REVIEW

The peer review history for this article is available at https://www.webofscience.com/api/gateway/wos/peer-review/10.1002/btm2.10535.

## Data Availability

The data that support the findings of this study are available from the corresponding author upon reasonable request.
